# Microbial interactions as key players shaping the emergence and spread of de novo resistance mutations

**DOI:** 10.1099/mic.0.001749

**Published:** 2026-08-11

**Authors:** Lauren Pittaccio, Jack Knowles, Rachel M. Wheatley

**Affiliations:** 1 School of Biological Sciences, Queen’s University Belfast, Belfast, UK

**Keywords:** antibiotic resistance, evolution, microbial interactions, microbiome, resistance mutations

## Abstract

Antibiotic resistance and the microbiome are two of the most prominent and highly active research areas currently in microbiology. However, these studies are commonly siloed. Research into antibiotic resistance often takes a highly pathogenic-centric view, and microbiome studies typically assess changes in community composition at the genus or species level, rather than at the level of small changes in bacterial genotype that often underpin rapid and significant changes in antibiotic resistance. One of the major mechanisms of antibiotic resistance evolution is via the acquisition of *de novo* resistance mutations, spontaneous mutations that occur randomly and provide a selective advantage in the presence of antibiotics. In this perspective, we address how interactions within the microbiome can shape the emergence and spread of *de novo* resistance mutations. We outline existing theoretical and empirical support for how microbial interactions have the potential to influence (i) the probability of *de novo* resistance mutations emerging, (ii) the fitness costs associated with new resistance mutations and (iii) the long-term selection against resistance and the ability of resistant mutants to transmit to new sites. Existing evolutionary theory may help us predict how microbial interactions will impact the probability of resistance mutations emerging, through understanding how microbial communities will impact pathogen population size, mutation rates and the supply of genetic variation. While a number of these links are simple and intuitive, there is a need for empirical data to understand how the complexity of interactions that exist within a microbial community at any one time come together to shape the evolution of antibiotic resistance. Future research in this field has the potential to inform the development of novel strategies to combat antibiotic resistance based on manipulating microbial interactions.

## Introduction

Bacterial pathogens rarely exist in isolation but will typically encounter broader microbial communities. This may be in the context of invading an infection site, existing as part of a complex polymicrobial infection community or with recovery of the niche microbiome following infection. Within these communities, microbes will interact through various direct and indirect mechanisms, including via signalling molecules, the exchange of metabolites or toxins, and competition for resources and space [1–3]. These interactions can be a major driving force of within-host bacterial evolution and play a key role in shaping the ability of an invading pathogen to colonise a new niche [3–5].

Our understanding of how antibiotics work is commonly derived from experiments on single-species bacterial cultures under laboratory conditions. While this research has elucidated fundamental mechanisms of antibiotic resistance [6–9], it falls short of representing the complexity of the biological environments where infections occur. For example, it is well established that the results from antibiotic susceptibility testing in the lab do not always predict the response in patients, and several recent studies have established that interspecies interactions within microbial communities can compromise antibiotic efficacy. This microbiome-mediated impairment to antibiotic efficacy may occur via the secretion of antibiotic deactivating enzymes from neighbouring cells [10–12], through alterations to metabolism and growth of a focal pathogen species [13], through upregulation of antibiotic efflux pump encoding genes [14] or via other physiological mechanisms leading to increased antibiotic tolerance [15]. Despite this body of work and a growing appreciation of the importance of microbial ecology during infections, there is currently limited empirical data on when and how microbial interactions may influence the evolution of antibiotic resistance.

One of the major mechanisms of resistance evolution is via the acquisition of *de novo* resistance mutations. These are spontaneous mutations that occur randomly within a previously susceptible population and provide a selective advantage in the presence of antibiotics. *Mycobacterium tuberculosis* is a pertinent, if extreme, example of their clinical significance, where antibiotic resistance evolves almost entirely by chromosomal mutations as opposed to horizontal gene transfer [16]. Common *de novo* resistance mutations include mutations in the targets of antibiotics, mutations that impair the function of porins utilized by antibiotics to get into the cell, and mutations that upregulate multidrug efflux pumps [17]. This type of rapid *de novo* resistance evolution can drive large changes in resistance over short-time scales and is a major problem for the emergence of resistance over the course of treating an infection [18–20].

In this perspective, we outline existing theoretical and empirical support for how the interactions between microbes have the potential to influence (i) the probability of *de novo* resistance mutations emerging, (ii) the fitness costs associated with new resistance mutations and (iii) the long-term selection against resistance and the ability of resistant mutants to transmit to new sites. Here, we define *de novo* resistance mutations as any type of spontaneous mutation that confers a selective advantage in the presence of antibiotics due to its ability to limit the inhibitory or killing action of an antibiotic. While we focus on mutational resistance, conceptually this framework could also be applied to the acquisition of resistance genes via horizontal gene transfer in many scenarios.

This perspective integrates recently published data with our own theoretical frameworks and extends the framework proposed by De Wit *et al*. [21]. In their framework, De Wit *et al*. defined the factors by which interspecies microbial interactions may influence resistance evolution via an effect on either the emergence of a resistant mutant or the spread of resistance through the population. We delineate the spread of resistance into two categories: the immediate fitness costs associated with a resistance mutation, and the long-term success and ability of the resistant mutant to transmit to new sites. Finally, we highlight several priorities for future research. Given the potential of the microbiome to shape the emergence and spread of *de novo* resistance mutations, future work in this field may allow us to devise novel strategies to combat antibiotic-resistant pathogens based on manipulating microbial interactions.

### The probability of *de novo* resistance mutations emerging

A foundational concept in evolutionary biology is that the adaptive potential of a population will be shaped by the contributions of standing genetic variation along with the generation of variation during selection [22]. Standing genetic variation is broadly understood to be the most important for fuelling rapid adaptation, and population size is a key parameter shaping the supply of genetic variation, in terms of both the standing genetic variation that will be available and the ability of that population to generate new mutations.

**Fig. 1.**
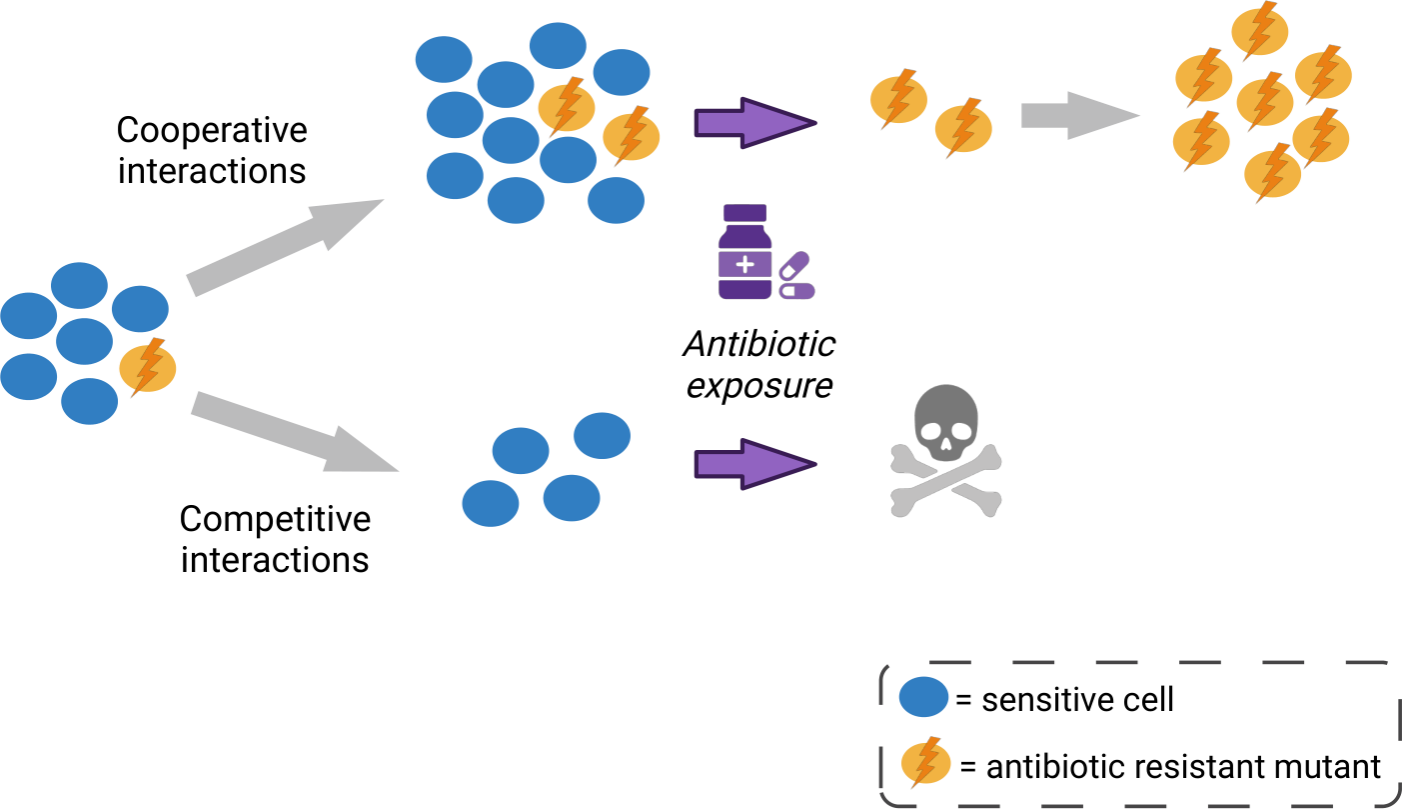
Conceptual diagram showing the impact of competitive or cooperative microbial interactions on the population size of a focal pathogen species and the likelihood that *de novo* resistance mutations may emerge. Figure created using BioRender.

As a simple but intuitive example of how microbial interactions may impact the probability of *de novo* resistance mutations emerging, competitive microbial interactions that suppress the growth of a focal pathogen will decrease population size and the supply of genetic variation, reducing the likelihood of new spontaneous resistance mutations emerging (Fig. 1). Competitive interactions may take place in the form of indirect competition, for example depletion of a shared resource, or as direct competition, for example, contact-dependent killing via the type VI secretion system [1, 23]. Competitive interactions are widespread in nature and play an important role in shaping community structure [3, 24–26]. Purely from the perspective of their ability to shape population sizes and community structure, competitive interactions likely play a pivotal role in the probability of *de novo* resistance mutations emerging.

As examples of empirical work in support of this, Adamowicz *et al*. [27] evidence that resistance to rifampicin and ampicillin evolves more slowly in co-culture rather than monoculture conditions with *Escherichia coli* and *Salmonella enterica* [27]. Similarly, work by Baumgartner *et al*. [28] shows that chromosomal resistance mutations in *E. coli* evolve only in the absence of resident microbial communities [28]. Phage-mediated interactions can also be a major determinant of population composition in microbial communities [29, 30], and through this impact on population size, phage may reduce the probability of *de novo* resistance mutations emerging via direct lytic killing. This effect of lytic phage has been demonstrated in experiments where the presence of a *Pseudomonas fluorescens*-targeting lytic phage in combination with antibiotics dramatically reduces the chance of *P. fluorescens* survival and antibiotic resistance emergence, compared to antibiotics in the absence of phage [31].

Cooperative bacterial interactions that positively impact the growth of a focal pathogen (for example, via cross-feeding) may act to the opposite effect and increase the likelihood of new spontaneous resistance mutations emerging (Fig. 1). For instance, *β*-lactamase-producing bacteria can enable survival of susceptible bacteria in a community [10, 11]. This increased survival and population size of the susceptible bacteria will increase the likelihood that *de novo β*-lactam-resistant mutants may arise. While this link between competitive or cooperative bacterial interactions and resistance mutations is intuitive and based on fundamental evolutionary theory, there are only a small number of examples dissecting this in action [10, 11, 27, 32]. How this trades off with molecular mechanisms of microbial interactions is unclear, and work has generally used low complexity bacterial communities consisting of a small number of microbes. While this has been used to leverage mechanistic insight, this generally is not representative of the complexity found in natural microbial communities.

Microbial interactions may also shape mutation supply via an impact on mutation rate. We have a few examples where this has been studied directly. For example, both *Pseudomonas aeruginosa* and *Candida albicans* display an increased mutation rate in co-culture compared to monoculture conditions [33], as do *P. aeruginosa* and *Staphylococcus aureus* [34]. Similarly, a number of environmental stress-related factors are known to impact mutation rate, such as oxidative stress [35] and resource limitation [36, 37], and these factors are also likely to be influenced by the presence or absence of different microbial community members.

### The fitness costs associated with new resistance mutations

The acquisition of *de novo* resistance mutations is often associated with a fitness cost, expressed in terms of reduced competitive ability in the absence of antibiotics [38–40]. Once the selective pressure of antibiotic treatment is removed, bacterial cells possessing this new resistance mutation may be outcompeted by their sensitive ancestors and lost from the population, in a manner depending on the magnitude of these fitness costs and the presence of sensitive ancestors surviving antibiotic treatment. These fitness costs are thought to represent one of the major obstacles to the spread of resistance at an epidemiological scale [39].

Fitness costs may originate through a number of mechanisms [38, 39]. For instance, resistance mutations that reduce porin expression and membrane permeability may simultaneously impair nutrient uptake [41, 42], and resistance mutations in ribosomal proteins may impose a fitness cost through reducing the rate of protein synthesis [43, 44]. Microbial interactions may impact the fitness costs associated with new resistance mutations by a number of theoretical mechanisms. For example, increased resource competition resulting from the presence of microbes with overlapping resource requirements may more drastically impact cells with an already impaired nutrient uptake due to reduced membrane permeability. In support of this, work by Klümper *et al*. [45] demonstrates that being embedded in a pig gut-derived microbial community increases the fitness costs associated with a gentamicin resistance mutation in *E. coli* [45]. This is suggested to be due to the impacts of resource limitation having a more pronounced effect on the resistant genotype [45]. Similarly, resistance mutations in ribosomal proteins reducing the rate of protein synthesis may slow the rate at which cells are able to respond to new stressors, such as may be imposed by the presence of competing microbes.

**Fig. 2.**
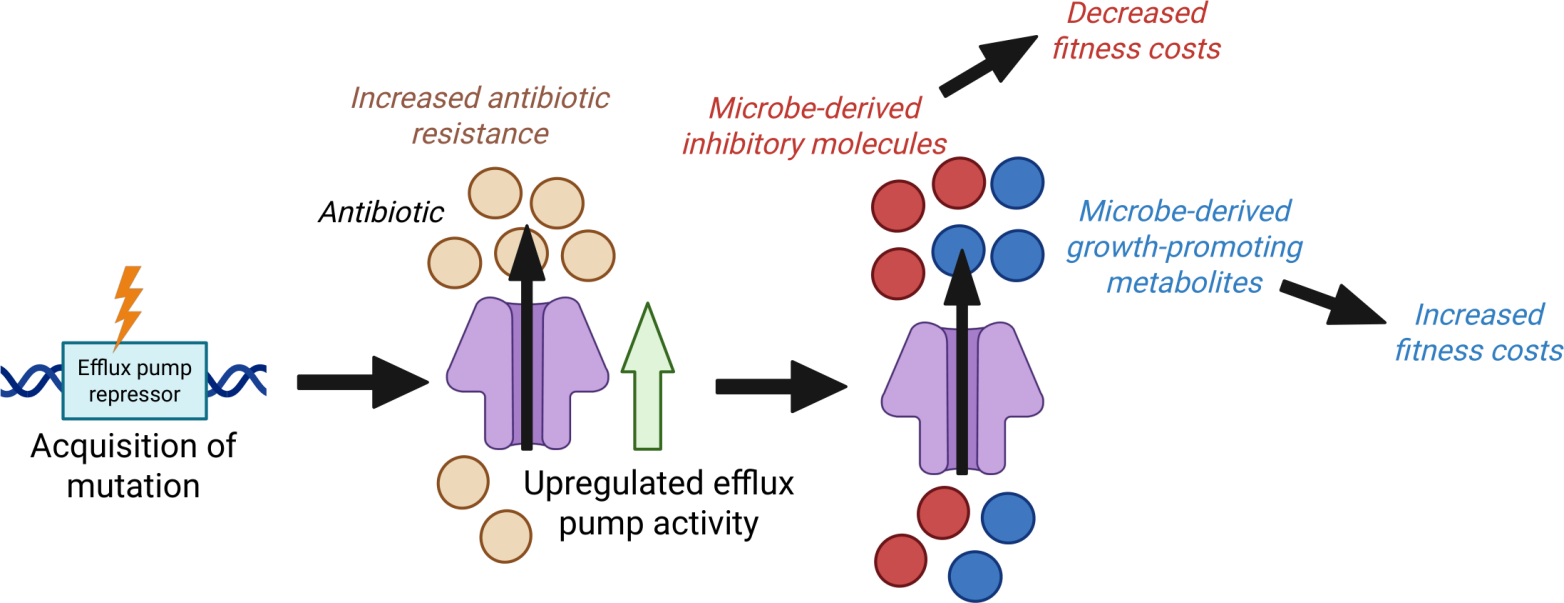
Hypothetical mechanism by which microbial interactions may lead to increased or decreased fitness costs associated with *de novo* resistance mutations in multidrug efflux pump regulatory genes. Acquisition of a non-synonymous mutation in a multidrug efflux pump repressor leads to loss of multidrug efflux pump repression and upregulated efflux pump activity. Upregulated efflux pump activity leads to increased antibiotic resistance, via antibiotic efflux, alongside efflux of both microbially derived inhibitory molecules (e.g. toxins) and microbially derived growth-promoting metabolites. Export of microbially derived inhibitory molecules will decrease the fitness costs associated with antibiotic resistance by providing a growth advantage to the resistant mutant in the presence of the producer microbe. Export of microbially derived growth-promoting metabolites will increase the fitness costs associated with antibiotic resistance by imposing a comparative growth penalty on the resistant mutant in the presence of the producer microbe. Figure created using BioRender.

It is important to consider how the mechanisms of antibiotic resistance may interface with the molecular mechanisms of microbial interaction. Multidrug efflux pumps can efflux several different substrates in addition to antibiotics, such as metals, dyes, detergents, bacterial metabolites and signalling compounds [46–48]. If these pumps efflux a molecule produced by neighbouring microbes, such as a metabolite beneficial for growth or an inhibitory toxin, the presence of the producing microbe will also shape the fitness costs of an efflux pump-resistant mutant (Fig. 2).

Previous work in our group has shown that resistance mutations in *P. aeruginosa* genes involved in multidrug efflux pump repression have reduced fitness costs in the presence of *Staphylococcus lugdunensis* but increased fitness costs in the presence of *Rothia mucilaginosa* and *Staphylococcus epidermidis* [49]. One mechanism that could explain this finding is that different molecules produced by these microbes are efflux pump substrates, and this is driving differential fitness costs in the efflux pump upregulator mutants (Fig. 2). *R. mucilaginosa* can help facilitate *P. aeruginosa* growth via cross-feeding [50], so we hypothesized that the decreased fitness of the efflux pump repressor mutants could be due to the increased efflux of *R. mucilaginosa*-derived metabolites that support *P. aeruginosa* growth [49]. While a number of studies such as this one have evidenced an impact of microbial community on fitness costs of antibiotic resistance mutations [45, 49, 51], there is a need for further detailed mechanistic work to reveal the molecular mechanisms underlying these changes to fitness costs.

### The long-term selection against resistance and the ability of resistant mutants to transmit to new sites

Long-term success of the resistant mutant and its ability to transmit to new sites will be shaped by the fitness of the strain and its ability to acquire additional genetic changes in the surrounding environment. In terms of ability to colonise a new site, recent work has shown that the success of antibiotic-resistant strains to invade gut microbiome communities is significantly impacted by the strain composition of these microbial communities [52]. This is determined by interactions at both the strain and species level [52]. Similarly, work in our lab has demonstrated that antibiotic-resistant mutants of *P. aeruginosa* differ in their ability to invade bacteria from the respiratory microbiome, in a genotype- and microbiome strain identity-dependent manner [49]. While it is well established that composition of the host microbiota is important in mediating colonisation resistance to pathogen invasion [3, 53, 54], what is interesting is that these relatively minor genetic changes associated with resistance (e.g. at the single nucleotide polymorphism level) can lead to profoundly different outcomes compared to the ancestral strain on the basis of these specific microbial interactions.

Similarly, any benefit or disadvantage in terms of cross-resistance or cross-sensitivity to microbially produced molecules present in the community will also shape selection of a resistant mutant over time. Many antibiotics were originally isolated from microbes or are modifications of microbial products and mediate inter-microbial competition in their natural capacity. If acquiring antibiotic resistance provides cross-resistance to molecules produced within a microbial community, resistance may also improve invasion ability into that community. Cross-resistance between antibiotics and bacteriocins has been reported [55–57], suggesting that some antibiotic-resistant mutants may see a selective advantage in certain bacteriocin-producing communities. This is significant because it implies that a strain may not only be more resistant to antibiotics, but it may be better equipped at invading other microbial communities too. Future work uncovering the scope of cross-resistance between clinical antibiotics and microbially derived products will help us better understand this risk.

### Conclusion and future directions

Rising antibiotic resistance is threatening our ability to successfully treat bacterial infections. Microbial interactions are a fundamental component of bacterial infections, and in this perspective, we outline the need for systematic empirical research on when and how microbial interactions impact the ability of *de novo* resistance mutations to emerge and spread in bacterial populations. We describe existing theoretical and empirical support for how microbial interactions have the potential to influence: (i) the probability of *de novo* resistance mutations emerging, (ii) the fitness costs associated with new resistance mutations and (iii) the long-term selection against resistance and the ability of resistant mutants to transmit to new sites. This impact of microbial interactions on resistance mutations can be mediated through ecological context and indirect mechanisms, such as via resource competition and niche overlap, or through direct mechanisms, such as the molecules exchanged in microbial interaction interfacing directly with resistance mechanisms. Existing evolutionary theory has a role in helping us predict how microbial interactions may impact the probability of *de novo* resistance mutations emerging, through understanding how microbial communities will impact pathogen population size, mutation rates and the supply of genetic variation. These links are simple and intuitive, but there is a need for empirical data to understand how the multitude of interactions that may be present in a microbial community at any one time come together to shape these factors and the evolution of antibiotic resistance.

We focus on spontaneous resistance mutations, but conceptually, this logic could also be applied to the acquisition of resistance genes via horizontal gene transfer in many scenarios. For example, how microbial interactions impact population size will impact both the probability of *de novo* resistance mutations emerging and the supply of potential recipient cells for the horizontal transfer of plasmid-borne resistance genes. Furthermore, how the mechanisms of antibiotic resistance interface with the molecular mechanisms of microbial interaction is likely equally important for the downstream selection of both mutationally acquired resistance and horizontally acquired resistance.

We highlight several priorities for future research. First, microbiomes and the interactions within them are complex. A pressing challenge in the field is to move from correlations and associations to mechanisms. We need to consider how mechanisms of antibiotic resistance will interface with the molecular mechanisms of microbial interaction. This will require detailed mechanistic work to better define the molecular mechanisms of microbial interaction within a microbiome and how this is impacted by intra- and inter-host variability of microbiome compositions and infection-niche environments. Second, much of the existing research has focused on using microbial communities that consist of just a small number of interacting microbes (e.g. two or three). While this provides benefits in terms of ease of manipulation and the ability to leverage mechanistic insight, this generally is not representative of the complexity found in natural microbial communities and excludes possibilities of understanding higher order interactions. Future research with more complex microbial communities, but utilising approaches such as a drop-out community approach in which single members of a community are systematically removed and replaced [58], could help bridge this gap and overcome some of these challenges.

Finally, given the multitude of theoretical mechanisms by which microbial interactions may impact resistance evolution, we need empirical data to understand which factors are most critical in determining evolvability (*impact on population size? molecular exchange? shared public goods? cross-resistance?*). Answering this question could be aided by systematic experimental work alongside complementary computational modelling approaches (e.g. [59]). This knowledge has the potential to inform the development of new strategies to combat antibiotic resistance based on manipulating microbial interactions. As an example of these principles in action, recent work by Tu *et al*. [60] demonstrated that bacteria expressing a metallo-*β*-lactamase had an elevated fitness cost in clinically relevant, zinc-deprived environments [60]. They showed that this cost can be exploited via fitness trade-off manipulation with the use of unconventional antibiotics to combat metallo-*β*-lactamase expressing pathogens [60]. While the costly environment in this example is host-mediated, this same logic has the potential to be applied to microbial interactions, by identifying microbes or microbial products that increase the fitness costs associated with resistance.
